# Antibacterial Silver Modified Gallium Metal-Organic Frameworks for Odontogenic Infections

**DOI:** 10.21203/rs.3.rs-6954848/v1

**Published:** 2025-08-06

**Authors:** Fellype Diorgennes Cordeiro Gomes, Diptomit Biswas, Mary Cristina Ferreira Alves, Severino Alves Júnior, Scott H. Medina

**Affiliations:** Federal University of Pernambuco, Cidade Universitária; Pennsylvania State University; Paraíba State University; Federal University of Pernambuco, Cidade Universitária; Pennsylvania State University

**Keywords:** Oral infection, Gallium, Silver Nanoparticles, Metal-Organic Frameworks, Antimicrobial

## Abstract

Surgical debridement of infected oral tissues can compromise maxillofacial aesthetics and anatomical function, thereby reinforcing the use of antibiotics as a first-line therapy. However, the escalating threat of multidrug-resistant pathogens has intensified the need for non-antibiotic alternatives that can be applied topically and eliminate microbes regardless of their resistance profiles. In this study, we introduce hybrid gallium–silver metal-organic frameworks (MOFs) as a novel therapeutic platform that integrates gallium-mediated disruption of iron-dependent metabolic pathways with the biophysical antimicrobial effects of silver nanoparticles, resulting in potent and rapid inhibition of oral streptococcal growth. Using complementary spectroscopic and microscopic techniques, we elucidate the structure and physicochemical properties of these gallium–silver MOFs, leveraging these insights to understand their antibacterial mechanisms. Cytotoxicity assays conducted on human osteogenic cells help define an appropriate therapeutic window. These proof-of-concept findings suggest that, with further optimization, gallium–silver MOFs hold promise as an attractive topical antimicrobial with potential to rapidly clear odontogenic infections.

## 1- INTRODUCTION

Odontogenic infections result from bacterial colonization of the teeth, gingival tissues, or adjacent bone structures.^1^ When not adequately treated, these localized infections can quickly evolve into more extensive maxillofacial conditions.^1^,^2^ Standard therapeutic approaches typically involve a combination of surgical debridement and systemic administration of broad-spectrum antibiotics. Although effective, these interventions carry significant limitations, including the risk of facial disfigurement or functional impairment following surgery and the emergence of antibiotic-resistant pathogens. These challenges underscore the urgent need for fast-acting, non-antibiotic therapies that can be locally administered, potentially reducing–or even eliminating–the necessity for surgical intervention.^3^,^4^

**Figure 1:**
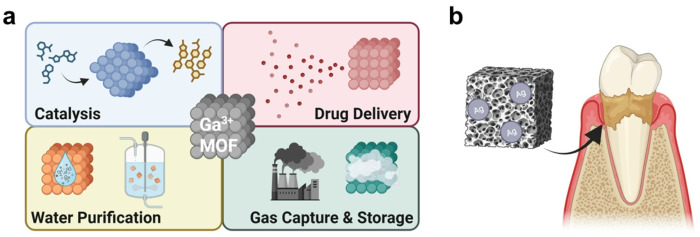
(**a**) Summary of the recent biomedical applications of Ga-MOFs. (**b**) Ga-MOFs functionalized with silver nanoparticles hold promise to rapidly clear dental infections, while promoting osteogenesis in the compromised oral tissues.

Towards this goal, antimicrobial and antioxidant nanoparticles represent a promising alternative to conventional antibiotics in dental disease.^5, 6^ Metallic nanoparticles, including silver, gold, copper and titanium based formulations, have attracted particular attention due to their instrinsic antimicrobial activity, anti-biofilm effects and photocatalytic properties.^7–10^ Among the array of available metals to choose from, Gallium (Ga), a post-transition metal with iron-like properties,^11–13^ has been comparitavely understudied, despite its potential to interfere with iron metabolism to inhibit bacterial growth. The additional osteoinducing effects of Ga^3+^ ions suggests this metal represents an ideal therapeutic scaffold to clear dental infections and promote bone regrowth.^12^ However, Ga’s rapid hydrolysis in tissues limits its solubility and bioavailability, ultimately prohibiting the clinical translation of its free ion form. To overcome this, Ga has been organized with various organic ligands into more hydrophilic crystalline lattices, referred to as metal-organic frameworks (MOFs).^13, 14^ MOFs exhibit a wide range of advantageous properties, including thermal and mechanical stability, tunable porous structures for compound storage, and aqueous solubility.^15–17^ These characteristics, together with an abundance of available inorganic and organic linkers, have enabled their use in biocatalysis, water purification, drug delivery and gas separation (Fig. 1a). Here, we combine the iron metabolism inhibitory activity of Ga-MOFs with antibacterial silver nanoparticles (AgNPs) to generate hybrid MOFs with potent antimicrobial effects towards the oral pathogens *S. Mitis* and *S. Pneumoniae*. Thorough structural and thermodynamic characterization studies elucidate the nature of Ga-MOF-AgNP interactions, and cell based assays demonstrate the biocompatability of the platform towards human derived bone cells. These findings support the continued development of Ag-modified Ga-MOFs as an attractive therapeutic platform for rapid clearance of dental and maxofacilial infections without inducing significant further osteopenia (Fig. 1b).

## 2- EXPERIMENTAL

### 2.1- Materials:

Gallium nitrate (Ga(NO_3_)_3_.XH_2_O), Polyvinylpyrrolidone (PVP), Silver nitrate (AgNO_3_), Mellitic acid (C_12_H_6_O_12_), Yeast extract, and MTT (3-(4,5-dimethylthiazol-2-yl)-2,5-diphenyl tetrazolium bromide) were purchased from Sigma-Aldrich. Sodium citrate (C_6_H_5_Na_3_O_7_.2H_2_O) was purchased from Sigma-Aldrich. Dimethyl sulfoxide (DMSO) was purchased from Fisher BioReagents, and Tryptic Soy broth purchased from MP Biomedicals. All reagents were of analytical grade and were used without any further purification.

### 2.2- Experimental procedure

#### 2.2.1- Synthesis of Ga-MIL-116 decorated with silver nanoparticles - (Ga-MIL-116@AgNPs)

Ga-MIL-116 was synthesized by the hydrothermal method, as described by Volkringer et al.^18^ The synthesis of silver nanoparticles was carried out based on the method of Lee and Meisel,^19^ and then adapted for gallium MOF. In brief, 125 mL of a silver nitrate (AgNO_3_) solution with a concentration of 0.001 M was heated in an Erlenmeyer flask until boiling, with magnetic stirring. At a temperature of 95°-100° C, Ga-MIL-116, prepared as a homogenous suspension in water (10 mg of Ga-MIL-116 in 1 mL of water) was added and kept stirring for 5 minutes. Using a Pasteur pipette, 1 mL of the reducing agent, sodium citrate (Na_3_C_6_H_5_O_7_), with a concentration of 0.038 M, was added at a rate of one drop per second. Subsequently, 10 drops of PVP previously dissolved in water (0.05 g of PVP in 5 mL of water) were added. A color change from yellow to amber was observed, confirming the formation of silver nanoparticles. Finally, samples were dried in an oven at 50° C.

#### 2.2.2- Antimicrobial susceptibility testing

The sensitivity of bacterial strains to the study materials (Ga-MIL-116@AgNPs) was tested using *S. Mitis* (ATCC 49456) and *S. pneumoniae* (unencapsulated type 2 D39 obtained from the National Collection of Type Cultures; NCTC). Both were grown in Trypticase Soy Broth (TSB) + 0.5% yeast extract by dilution method in 96-well microplate.^20^ The gallium MOF samples decorated with/without silver nanoparticles were suspended in 2% DMSO in TSB with an initial/stock concentration of 2000 μg/mL, of which 50 μL was deposited in 96-well microplates containing 50 μL of TSB culture medium + 0.5% yeast extract. Bacterial growth was measured via OD_600_ and the cultures were diluted to an OD_600_ value of 0.02 before adding it to the treated wells in a 1:1 v/v ratio. Blank broth or 20% DMSO were used as negative and positive controls, respectively. A microplate reader (Synergy H1 Biotek microplate reader, USA) at 600 nm was used to collect time dependent growth data. Antimicrobial tests were performed in triplicate.

#### 2.2.3- Cytocompatibility tests

The MTT assay^21^ was carried out to evaluate the effect of Ga-MIL-116@AgNPs on cell viability. Biological tests were carried out using MG-63 human osteosarcoma (originating from bone tissue - Rio de Janeiro Cell Bank, Rio de Janeiro, Brazil). These cells were cultured in Dulbecco’s modified Eagle’s medium (DMEM), supplemented with 10% fetal bovine serum (FBS), L-glutamine (2 mM) and penicillin/streptomycin (1%), in a humidity-controlled greenhouse and atmosphere containing 5% CO_2_, at a temperature of 37°C. Initially, cells were cultured in a 96-well plate, at a density of 3 x 10^3^ cells/well, for 20 hours. Then, the cells were treated with different concentrations of the samples (3.0; 6.0; 12.0; 25.0; 50.0 or 100 μg/mL) for a period of 24 hours. After the incubation period with the treatment, 10 μL of MTT, dissolved in DMEM at a concentration of 5 mg/mL, was added to each well for a period of 3 hours. Subsequently, the supernatant was discarded and 150 μL of DMSO was added to each well to solubilize the formed formazan crystals. Using a microplate spectrophotometer, the absorbance of each well was recorded at a wavelength of 540 nm, thus measuring the optical density (OD). The percentage of cell viability was calculated using the formula: (OD of treated cells / OD of untreated cells) x 100.

### 2.3- Characterization Experiments

The crystalline structure of Ga-MIL-116@AgNPs was analyzed using powder X-ray diffraction (PXRD). Measurements were performed on a SmartLab diffractometer (Rigaku, Japan) with Cu Kα radiation (λ = 1.5406 Å). The diffraction patterns were collected over a 2θ range of 5–80°, with a scanning step size of 0.01°/min. UV–Vis absorption spectra were recorded on a Shimadzu UV-2600 spectrophotometer equipped with a diode array detector and integrating sphere. Measurements were conducted in the 200–800 nm wavelength range, with a spectral resolution of 1 nm, at 25°C. Thermal behavior was evaluated using a Shimadzu DTG-60H thermobalance. Samples were placed in platinum pans and heated from room temperature to 900°C at a rate of 10°C/min under a nitrogen flow of 100 mL/min. FTIR spectra were acquired using a PerkinElmer Spectrum 400 spectrometer (Serial No. 82287) in the range of 4000–400 cm^−1^. Samples were analyzed in the solid state using ATR mode. TEM imaging was performed to evaluate the morphology and spatial distribution of silver nanoparticles within the MOF matrix. Samples were prepared via drop-casting onto lacey carbon-coated copper grids and analyzed using a FEI Talos transmission electron microscope operating at 200 kV.

## 3- RESULTS AND DISCUSSION

**Figure 2:**
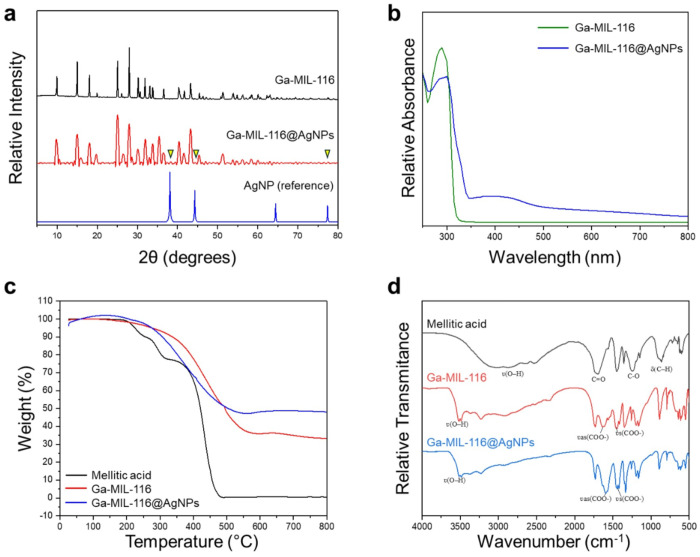
(**a**) Diffractograms of Ga-MIL-116 and Ga-MIL-116@AgNPs, relative to the silver nanoparticle reference (AgNP). Yellow arrows highlight AgNP peaks in the Ga-MIL-116@AgNP spectra. (**b**) UV-Vis spectra of Ga-MIL-116 and Ga-MIL-116@AgNPs. (**c, d**) Thermogravimetric curves (c) and infrared absorption spectra (d) of Ga-MIL-116 and Ga-MIL-116@AgNPs. The organic linker mellitic acid is included as a reference control.

X-ray diffraction (XRD) analysis of the Ga MOF composite (Ga-MIL-116) suggests an orthorhombic three-dimensional configuration for the base scaffold (Fig. 2a). Addition of silver nanoparticles (Ga-MIL-116@AgNPs) showed a similar peak pattern with reduced overal reflection intensity compared to Ga-MIL-116, which is attributed to the deposition of silver nanoparticles on the surface of the porous MOF structure.^22^ The XRD pattern of Ga-MIL-116@AgNPs additionally revealed diffraction peaks at 2θ = 38.44°, 44.62°, and 77.56°, which correspond to reflections from the (111), (200), and (311) lattice planes consistent with the face-centered cubic structure of silver in AgNPs reported from reference standards.^19^ Yet, these silver-specific XRD features are relatively weak compared to the MOF scaffold. Therefore, we additionally performed UV/Vis spectrophotometry to interpret the optical properties of the structures and confirm the presence of bound silver ions (Fig. 2b).^22^ Spectra collected for the bare Ga-MIL-116 MOF showed a characteristic peak at 295 nm, corresponding to the π-π* transitions of the organic mellitic acid linker. This feature was diminished following AgNP complexation, and a broad peak spanning 350–500 nm emerged, confirming the presence of silver bound to the MOF scaffold.

Next, thermogravimetric analysis (TGA) was performed to investigate the temperature-dependent mass variation of the samples to infer stability properties and water content. Figure 2c shows the mellitic acid ligand, as a control, begins to decompose at 214°C, with a 90% mass loss observed at 476°C. The TGA profile for Ga-MIL-116 reveals a slight reduction in mass (~ 4.0%) at 200°C that suggests libration of bound water molecules^23^. Between, 250°C and 550°C the coordinating MOF linker thermally decomposes, leading to an overall 57% mass loss. The introduction of silver nanoparticles resulted in a moderate increase in the thermal stability of the Ga-MIL-116@AgNP composite, with a shift of the mass loss onset point to 260°C and total mass loss of 47% at > 500°C. Taken together, this suggests that AgNPs bound onto, and within, the porous MOF scaffold serve to enhance the thermal stability of the complex.

Finally, infrared spectroscopy was used to interrogate the interactions between Ga^3+^ ions, organic linkers and AgNPs, by interpreting the vibrational symmetric (ʋs) and asymmetric (ʋa) stretching frequencies of the different materials.^23^ To interpret compound interactions, mellitic acid was first analyzed as a reference and showed characteristic broad ʋ(O–H) stretching vibrations of it’s protonated carboxylic groups centered at 2862 cm^−1^. Intense bands at 1697 cm^−1^ and 1255 cm^−1^ are attributed to carboxy C = O and C–O stretching, respectively. In addition, the band at 863 cm^−1^ is the result of out-of-plane angular deformations of C–H bonds within the aromatic ring. These results are in agreement with what is expected for aromatic carboxylic acids, as reported by Silverstein.^24^ Utilizing this spectra as a reference, Ga-MIL-116 was next analyzed and showed a shift of mellitic acid’s symmetric ʋs(COO−) and asymmetric ʋas(COO−) carboxyl stretching to 1453 cm^−1^ and 1639 cm^−1^, respectively, suggesting coordination of the organic linker with gallium ions. These bands further shifted to lower wavenumbers in the presence of AgNPs (1440 and 1590 cm^−1^), indicating multivalent interactions of the linker with both the Ga^3+^ scaffold and bound silver. Both Ga-MIL-116 and Ga-MIL-116@AgNP also showed an absence of the mellitic acid C = O stretching reference band centered at 1697 cm^−1^, supporting altered resonance of the carboxyl group due to ion coordination.^25^ An additional absorption band centered at 3521 cm^−1^ observed for both structures is ascribed to coordinating water molecules.

**Figure 3:**
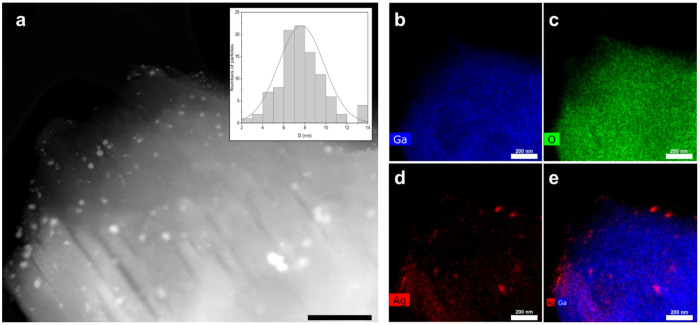
Transmission Electron Microscopy-Energy Dispersive X-ray Spectroscopy (TEM-EDS) imaging of Ga-MIL-116@AgNPs. (**a**) High-angle Annular Dark-field image. Scale bar = 200 nm. *Inset*: Frequency histogram of AgNP cluster diameter asdorped to the surface of Ga-MIL-116 MOFs. (**b– e**) Gallium (b), oxygen (c) and silver (d) elemental mapping, as well as the merge of the gallium and silver elemental signals (e).

Next, transmission electron microscopy with energy dispersive X-ray spectroscopy (TEM-EDS) was performed on Ga-MIL-116@AgNPs to visualize the elemental distribution within the MOF structure. The High-Angle Annular Dark-field (HDAAF) micrograph shown in figure 3a highlights the crystalline structure of the MOF scaffold, with distinguishable silver clusters approximately 4 – 10nm in diameter bound to the surface. As expected, Gallium (Ga) was homogenously distributed throughout the MOF matrix (Figure 3b), and overlayed with the oxygen (O) signal from the mellitic acid linker (Figure 3c). The Ag signal (Figure 3d), in addition to its superposition with Ga (Figure 3e), suggests that the silver particles are superficially deposited after the formation of the Ga-MIL-116 structure, or that they were partially incorporated into the pores during synthesis, acting as active centers to exert antimicrobial activity.

**Figure 4:**
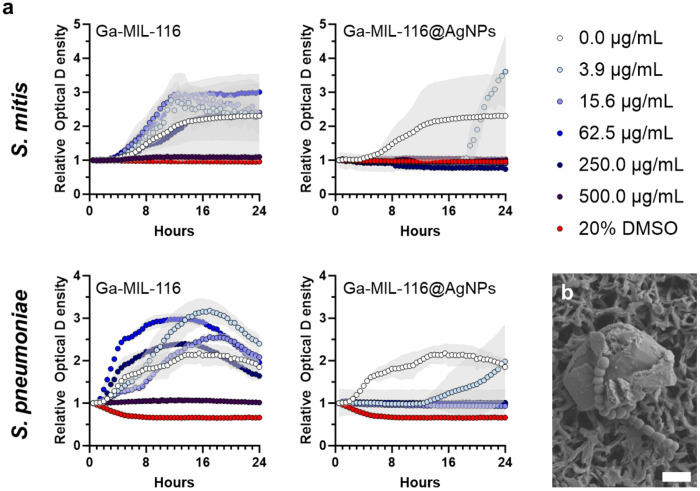
(**a**) Time-dependent relative optical density of *S. Mitis* (top) and *S. Pneumoniae* (bottom) microbes grown in the presence of Ga-MIL-116 (left) or Ga-MIL-116@AgNPs (right). Untreated (0.0 μg/mL) and 20% DMSO treated samples included as negative and positive controls, respectively. Standard deviations of the curves are shown by the respective light gray colored overlay. The complete concentration series can be found in Supplementary Figure S1. (**b**) Scanning electron micrograph of *S. Pneumoniae* interactions with a Ga-MIL-116@AgNP crystalline MOF. Scale bar = 2 μm.

The antibacterial synergy of Ga^3+^ and Ag^+^ ions was next tested in the model oral pathogens *S. mitis* and *S. pneumoniae* following treatment with silver deficient Ga-MIL-116 and silver containing Ga-MIL-116@AgNPs MOFs (Fig. 4a). Time-dependent optical density based growth studies demonstrate that the base Ga-MOF scaffold (Ga-MIL-116) is weakly active towards both microbes, with growth inhibition only observed at the highest tested concentration (500 μg/mL). In fact, concentrations of Ga-MIL-116 below 250 μg/mL appeared to marginally promote bacterial growth over the untreated control (0 μg/mL). This suggests that either the stability of the MOF structure prevents significant release of inhibitory Ga^3+^ ions over the 24 hours incubation period, or that released ions are not sufficiently potent to disrupt iron-dependent metabolism in the pathogen; or both. These observations rationalized the addition of antibacterial AgNPs to the MOF scaffold to enhance its activity. Bacteriologic testing demonstrated, as expected, the Ga-MIL-116@AgNPs MOFs showed strong antibacterial efficacy, with 3.9 μg/mL concentrations delaying bacterial outgrowth of both test microbes by ~ 10 hours and 7.8 μg/mL causing complete growth inhibition (complete concentration series can be found in Supplementary Figure S1). Additional scanning electron microscopy demonstrated that the streptococcal pathogens come into direct contact with the crystalline MOF structure to exert antimicrobial activity (Fig. 4b). The absence of visible morphologic changes to the contacting coccus in the collected images suggest that release of inhibitory silver ions, or the generation of reactive oxygen species, may potentiate antibacterial activity. These findings suggest that clustering AgNPs at the surface of Ga-MOFs can exert rapid and potent clearance of oral infections, positioning silver functionalized Ga-MOFs as a promising therapeutic alternative to conventional antibiotics.

**Figure 5:**
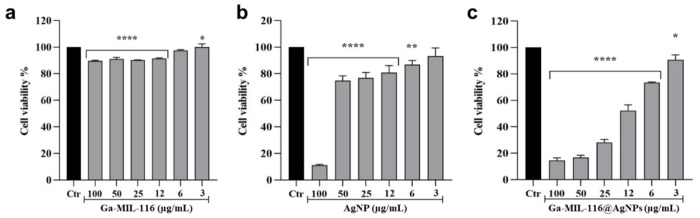
Viability of MG-63 human osteosarcoma cells treated with varying concentrations of (**a**) Ga-MIL-116, (**b**) silver nanoparticles (AgNP) or (**c**) Ga-MIL-116@AgNPs. Statistical significance determined relative to untreated control (Ctr, black) using one-way ANOVA followed by Tukey’s post hoc test, with * p < 0.05, ** p < 0.01, *** p < 0.001, and **** p < 0.0001.

Finally, we evaluated the cytotoxicity of the materials again MG-63 human osteogenic cells using colorimetric viability assays. Ga-MIL-116 were found to be generally well tolerated, with > 85% viability maintained after a 24 hour incubation at concentrations up to 100 μg/mL (Fig. 5a). Conversely, free AgNPs led to more significant off-target effects, with concentrations > 25 μg/mL reducing viability below 80% (Fig. 5b). Combining the two materials to produce Ga-MIL-116@AgNPs led to additive toxic effects, with concentrations of 12 μg/mL reducing cell viability ~ 50% (Fig. 5c). Given that Ga-MIL-116@AgNPs were effective against the streptococcal pathogens at concentrations at or above 3.9 μg/mL this suggests there is a narrow therapeutic window in which these compounds could be clinically effective as an oral therapeutic without harming host tissue. It is important to mention that MG-63 is an osteosarcoma cell line, and Ga has well documented antineoplastic effects against malignant cells due to disrupting iron-depedent metabolic processes.^26, 27^ Thus, the toxicity measured using this cancerous cell line may not be reflective of the sensitivity of non-malignant human osteocytes, warranting futher safety studies.

## 4- CONCLUSION

Metal nanoparticles continue to emerge as promising platforms for the development of topical oral anti-infective agents and antimicrobial dental materials. In this work, we report that hybridization of gallium-based metal—organic frameworks (Ga-MOFs) with silver nanoparticles (AgNPs) yields a multifunctional material—Ga-MIL-116@AgNPs—with rapid and potent activity against oral streptococci, alongside improved solubility and dispersion of the silver phase. Initial in vitro assessments indicate acceptable cytocompatibility at antibacterial doses; however, future studies should focus on evaluating biocompatibility in primary, non-malignant human osteogenic cells to better define the therapeutic window. Furthermore, rational surface passivation strategies aimed at controlling the release of Ag^+^ and Ga^3+^ ions may enhance safety and clinical applicability. Taken together, these results position gallium–silver MOF hybrids as a promising class of topical antimicrobials for the localized management of odontogenic infections, warranting further preclinical investigation.

## Supplementary Material

This is a list of supplementary files associated with this preprint. Click to download.

• SIMOFGaFellype.docx

## Data Availability

The data supporting this article have been included as part of the Supplementary Information, or are available upon request from the authors
